# Cumulative exposure to ADHD medication is inversely related to hippocampus subregional volume in children

**DOI:** 10.1016/j.nicl.2021.102695

**Published:** 2021-05-07

**Authors:** Nellie H. Fotopoulos, Gabriel A. Devenyi, Stephanie Guay, Sarojini M. Sengupta, M. Mallar Chakravarty, Natalie Grizenko, Sherif Karama, Ridha Joober

**Affiliations:** a Douglas Mental Health University Institute, Montréal, Québec, Canada; b Department of Human Genetics, McGill University, Montréal, Québec, Canada; c Department of Psychiatry, McGill University, Montréal, Québec, Canada; d Faculty of Dentistry, McGill University, Montréal, Québec, Canada; e Department of Neurology and Neurosurgery, McGill University, Montréal, Québec, Canada; f Montréal Neurological Institute, Montréal, Québec, Canada; g Department of Biological and Biomedical Engineering, McGill University, Montréal, Québec, Canada

**Keywords:** ADHD, Psychostimulants, Neuroimaging, Hippocampus, Children

## Abstract

**Background:**

Although there is some evidence for a normalization of brain structure following exposure to ADHD medication, literature on the effects of duration and dose of continued use on the brain is scarce. Here, we investigated the association between cumulative exposure to medication (range 1 week to 4.69 years) and cortical structures and subcortical volumes in a clinical sample of children with ADHD taking medication (n = 109). To the best of our knowledge, this is the first structural MRI study investigating the effects of cumulative exposure to medication on subregional volumes in children treated for ADHD.

**Methods:**

Cumulative exposure to ADHD medication (CEM) was defined as the product of duration on medication (days) and dose (mg/day), yielding the area under the curve (total mg). Cortical thickness and surface area measurements (CIVET-1.1.12), and subcortical volumes in 51 regions (MAGeT-Brain) were analyzed using general linear modelling.

**Results:**

Significant effects of CEM were found in two subregions of the left hippocampus, the CA1 (df = 95; q = 0.003) and the strata radiatum/lacunosum/moleculare (df = 95; q = 0.003). Specifically, higher CEM was associated with smaller volumes within these subregions. No effects of medication exposure were detected on cortical thickness or surface area.

**Conclusions:**

Although this study is cross-sectional, the results found within this sample of children show that prolonged ADHD medication use at higher doses is significantly associated with smaller hippocampus volumes in specific subregions. More research is required to determine whether these results are reproduced in other samples of children of ADHD, and further, whether these are beneficial or off-target effects of the medication.

## Highlights

• Cumulative exposure to ADHD medication characterized as the product of lifetime duration and dose.
• Medication effects investigated on 51 subregional volumes.
• Smaller hippocampus CA1 volumes associated with higher medication exposure.
• Effects remained when correcting for age and ADHD symptom severity.
• No global effects of medication on cortical thickness or surface area detected.

## 1 Introduction:

Attention deficit/hyperactivity disorder (ADHD) is the most common neurodevelopmental psychiatric disorder affecting 5–7% of children. The onset of ADHD occurs during childhood with core symptoms consisting of inattention, hyperactivity, and impulsivity (Faraone et al., 2015). Untreated ADHD has negative outcomes for the individual, family and society as it is associated with interpersonal conflicts, decreased self-esteem, poor academic performance, loss of productivity, traffic violations and premature/accidental death (Faraone et al., 2015, Franke et al., 2018, Klein et al., 2012).

Several studies have proposed that ADHD symptoms are associated with reduced activity in dopamine (DA) and norepinephrine (NE) systems (Faraone, 2018, Faraone et al., 2015). Changes in these neurotransmitter systems affect the functioning of brain structures relevant for ADHD (Faraone, 2018, Faraone et al., 2015). Psychostimulants such as methylphenidate (Ritalin©, Concerta© and Biphentin©) and amphetamines (Vyvanse© and Adderall©), as well as NE-specific therapeutic agents such as atomoxetine (Strattera©) increase DA/NE synaptic concentrations and have been shown to alleviate ADHD symptoms (Atkinson and Hollis, 2010, Briars and Todd, 2016, Rubia et al., 2014). It is estimated that 5.2% of children are taking ADHD medication, representing a five-fold increase from 1994 to 2010, as reported by the Center for Disease Control and in a 2018 study (Danielson et al., 2018, Prevention, 2018). The prominent increase in pharmacological treatment of ADHD may be partly attributable to the well-established positive effects on clinical outcomes and high efficacy, as 75 to 80% of children taking psychostimulants respond well (Atkinson & Hollis, 2010). Furthermore, ADHD is a treatable yet chronic disorder, and 50–60% of the children diagnosed with ADHD will have persistent symptoms into adulthood, thereby requiring ADHD medication across the lifespan (Faraone et al., 2015). As such, the typical course of treatment for ADHD involves continued administration of medication. However, human studies have primarily investigated the acute effects of ADHD medication between treatment-naïve and treatment-exposed children with ADHD. Consequently, the effects of long-term medication use on brain structure, remain to be clearly determined (Oakes et al., 2018, Schmitz et al., 2017).

Structural neuroimaging studies comparing ADHD children to neurotypical children identified group differences in cortical and subcortical brain regions (Albajara Saenz, Villemonteix, & Massat, 2018). A landmark study by Shaw et al. 2007 reported a delay in peak cortical maturation of 3.5 years in children with ADHD, most apparent in prefrontal regions (Shaw et al., 2007). A mega-analysis by Hoogman et al. 2017 reported reduced volumes in the accumbens, amygdala, hippocampus, putamen, and overall brain in comparison to control children (Hoogman et al., 2017). However, there is considerable variability across neuroimaging studies in ADHD, as one *meta*-analysis found that only 25–50% of published reports had reproducible results (Frodl & Skokauskas, 2012). Since pharmacological agents are commonly used to treat ADHD symptoms, it is important to assess their impact on brain structure. If exposure to ADHD medication significantly alters brain structure measurements, it might provide partial explanation for the varying results across ADHD imaging studies.

Some neuroimaging studies have investigated the effects of ADHD medication on brain structure. Previous studies generally compared three groups of children: medication-naïve with ADHD, treated with ADHD and unmedicated children with neurotypical development (control). One study reported that medicated children with ADHD did not significantly differ from control children with regards to cortical thickness. Though, a higher rate of cortical thinning was detected in unmedicated children with ADHD (Shaw et al., 2009). An earlier study by Castellanos et al. 2002 found that unmedicated children with ADHD had significantly smaller frontal, temporal and total white matter volumes in comparison to medicated and control children. However, they also reported children with ADHD had reduced total cortical grey matter volume regardless of their medication exposure, and concluded that grey matter, as opposed to white matter, may not be susceptible to medication effects (Castellanos et al., 2002). Other studies have observed significantly smaller white matter, anterior cingulate cortex, cerebellar and thalamic volumes in treatment-naïve children with ADHD relative to medicated and typically-developing children (Ivanov et al., 2010, Schweren et al., 2013, Semrud-Clikeman et al., 2014). Nevertheless, the longitudinal study by Shaw et al., reported no effects of ADHD medication on the basal ganglia (caudate, putamen and globus pallidus). The latest *meta*-regression analyses that identified brain differences between ADHD subjects and controls found no evidence of medication effects on brain structure (Hoogman et al., 2017, Hoogman et al., 2020). Specifically, correcting for exposure to medication did not alter main results. Limited data was available for medication use (only 44% had data available for exposure to medication), and as stated by the authors, the study had limited ability to investigate the role of medication use on the identified brain regions (Hoogman et al., 2020). Other studies investigated the effects of medication by comparing ADHD to subjects with OCD (Norman et al., 2016) and ASD (Lukito et al., 2020). Both studies reported no association between stimulant use and whole grey matter volumes(Norman et al., 2016) or ventral medial prefrontal cortex grey matter volume (Lukito et al., 2020). The only prospective longitudinal study reported an interaction effect (group and time) on left putamen grey matter volumes between the unmedicated group and the other two groups (medicated and controls). A volume decrease was noted in left putamen volumes in the non-medicated group compared to both the medicated group and controls, and no differences between the medicated group and controls, thereby suggesting a normalizing effect of medication (Pretus et al., 2017). Taken together, these studies do not provide evidence for abnormal brain development following exposure to ADHD medication. Rather, they highlight the confusing state of the literature where medication is reported as having either no effect on brain structure or as having a normalizing effect brain structure (Bledsoe et al., 2009, Loureiro-Vieira et al., 2017, Nakao et al., 2011).

Although normalization of certain brain structures by ADHD medication has been proposed, three important caveats should be considered. First, most studies assign a categorical designation (naïve vs. medicated) to investigate ADHD medication effects on the brain. Therefore, the effects of duration and dose of ADHD medication on brain structure in chronically-treated children have seldom been investigated. Indeed, neither of the two longitudinal studies nor the one prospective longitudinal study assessing the effects of ADHD medication on brain structure considered the duration or dose of medication use (Castellanos et al., 2002, Pretus et al., 2017, Shaw et al., 2014). Therefore, if a normalizing effect is occurring, the duration and dose required to achieve this remains unknown. There is a scarcity of studies using an accurate and continuous value for cumulative medication exposure to address this gap in the literature. The two studies currently available found no association when investigating the effects of cumulative medication intake on brain volumes (Greven et al., 2015) and cortical structures (Schweren et al., 2015).

Second, although the abovementioned studies report no medication effects on the brain regions investigated, other studies have reported hippocampus volume reductions in adults with ADHD who had, during childhood, been treated with ADHD medication (Frodl and Skokauskas, 2012, Onnink et al., 2014). These findings were not observed in stimulant-naïve adults with ADHD. Frodl and Skokauskas (2012) have suggested that changes in smaller regions, such as the hippocampus, may go undetected as large threshold corrections for the whole brain are typically used (Frodl & Skokauskas, 2012). Moreover, in the relatively few studies that have included the hippocampus when assessing medication effects, no studies have sought to investigate subregions.

Third, studies have documented the impact of motion in MRI research and have issued a note for caution when interpreting findings. Specifically, head and breathing motions during MRI acquisition led to underestimation of brain structure measurements (Reuter et al., 2015, Weinberger and Radulescu, 2016). Since children with ADHD tend to be hyperactive and pharmacological treatment reduces hyperactivity, it is possible that unmedicated children with ADHD have a significantly higher degree of motion during scanning and accrue more motion artifacts on raw brain images compared to medicated and control children. In this case, motion may confound the structural findings cited above and partly explain the observations of normalization (Pardoe, Kucharsky Hiess, & Kuzniecky, 2016). In combination to appropriate quality control, restricting the sample to children all undergoing pharmacological treatment for ADHD is one method to address this issue, as it removes the variability of motion that can arise between treatment groups.

The goal of this study is to examine the effects of cumulative exposure to ADHD medication (duration × dose) on cortical and subcortical brain structures in a clinical sample of children being treated for ADHD. The first objective is to determine the relationship between ADHD medication and cortical thickness and surface area. Since medication has been proposed in some studies to normalize brain structure measurements, we hypothesize that significant increases in mean cortical thickness and surface area are associated with cumulative exposure to ADHD medication (CEM). The second objective is to explore the effects of medication on the volume of 51 subregions within subcortical structures (i.e., cerebellum, hippocampus, striatum, thalamus and globus pallidus). To keep in line with the concept of normalization, we hypothesize that CEM is significantly correlated with subcortical volumes. To the best of our knowledge, this is the first structural MRI study investigating the effects of CEM (duration X dose) on 51 subregional volumes in a clinical sample of children with ADHD.

## 2 Methods:

### 2.1 Participants

One-hundred and forty-four unrelated children aged between 6 and 12 years (mean = 9.3 years, SD = 1.8) were recruited for the MRI study at the ADHD clinic of the Douglas Mental Health University Institute (DMHUI) in Montreal. The research protocol was approved by the Research Ethics Board of DMHUI. The study was explained to parents who provided written consent. Children gave verbal assent. Parents completed a general information questionnaire to acquire demographic data. Sample characteristics are available in Table 1.

**Table 1:** Sample demongraphics for ADHD and typically-developing children.

	ADHD (n=101)	Control (n=35)	Total (n=136)	Stats
Age yrs. (SD)	9.51 (1.67)	8.83 (2.1)	9.34 (1.8)	F_1,135_=3.88; *p*=.051
Full Scale IQ (SD)	97.26 (13.52)	109.94 (14.31)	100.52 (14.76)	F_1,131_=21.57; ***p*=.000**
Sex (% male)	76/101 (75)	16/35 (46)	92/136 (68)	X^2^=10.38; df=1; ***p*=.001**
Income (%)				
<10K	4/97 (4)	0/33 (0)	4/130 (3)	X^2^=1.69; df=2; *p*=.430
10-40K	21/97 (22)	6/33 (18)	27/130 (21)	
40K +	72/97 (79)	27/33 (82)	99/130 (76)	
Ethnicity (% Caucasian)	89/101 (88)	26/34 (77)	115/135 (85)	X^2^=2.74; df=1; *p*=.098
Handedness (%)				
Right	84/100 (84)	33/35 (94)	117/135 (87)	X^2^=2.39; df=2; *p*=.303
Left	7/100 (7)	1/35 (3)	8/135 (6)	
Ambidextrous	9/100 (9)	1/35 (3)	10/135 (7)	
Conner’s Total Baseline Parent (SD)	72.37 (10.89)	48.09 (5.16)	66.03 (14.45)	F_1,133_=160.77; ***p*=.000**
Conner’s Total Baseline Teacher (SD)	66.87 (11.56)	N/A	N/A	N/A
CBCL Total T-Score (SD)	67.70 (7.52)	44.15 (8.78)	61.71 (12.93)	F_1,133_=228.10; ***p*=.000**
DISC Total ADHD items (SD)	12.92 (3.32)	1.71 (1.92)	10.01 (5.78)	F_1,134_=355.34; ***p*=.000**

Bold indicates significant statistical difference between ADHD and control groups.

Out of the total sample (n = 144), 109 children had a confirmed diagnosis of ADHD based on a clinical evaluation by a psychiatrist according to Diagnostic and Statistical Manual of Mental Disorder version 4 (DSM-IV; 1994 version) criteria and corroborated with the Diagnostic Interview Schedule for Children (DISC) administered to parents (Diagnostic and Statistical Manual of Mental Disorders., 1994, Kasius et al., 1997). Parents completed the child behavioural checklist (CBCL) to acquire behavioural dimensions related to ADHD (e.g., anxiety, aggression, etc.). Information on the child’s behaviour in home and school settings was collected via the Conners’ Global Index scale from parents and teachers, respectively(Conners, Sitarenios, Parker, & Epstein, 1998). Children completed the Continuous Performance Test (CPT) to acquire measures of attention, impulse-control and response-inhibition (Conners, 1985). Children with an IQ less than 70 according to the Weschler Intelligence Scale for Children IV (WISC-IV), a diagnosis of Tourette syndrome, pervasive developmental disorder and/or psychosis, were excluded. A subgroup of matched typically-developing children was used as a control group for complementary analyses (n = 35).

### 2.2 Image acquisition

All children (n = 144; ADHD n = 109; control n = 35) were scanned on site at the Cerebral Imaging Center in a 3 T Siemens MRI scanner to acquire T1 and T2-weighted structural images. Scanning time consisted of two rounds of 9 min. The protocol was tailored to a pediatric population to reduce motion during scanning. All children practiced on a mock scanner prior to MRI scanning, a cartoon was shown, sandbags were placed over their extremities and scans were repeated when necessary. Protocol details are described elsewhere (Sengupta et al., 2018).

### 2.3 Image processing

An initial quality control was carried out to select a single optimal scan for every child, and two participants with ADHD were excluded due to motion (n = 107). Pre-processing of raw scans was conducted to minimize downstream failures via the minc-bpipe-library pipeline (https://github.com/CobraLab/minc-bpipe-library). Pre-processed scans were input to CIVET-1.1.12 and MAGeT-Brain for cortical and subcortical analysis, respectively. CIVET is an automated imaging software tool used to obtain corticometrics (version 1.1.12, Montreal Neurological Institute, McGill University, Montreal Quebec, Canada) (Ad-Dab'bagh et al., 2006, Collins et al., 1995, Sled et al., 1998). Cortical thickness and surface area were calculated at roughly 82 000 points across the cortex and data was blurred using the default surface-based diffusion kernel of 20 mm for thickness and 40 mm full-width at half-maximum for surface area. MAGeT-Brain was used to extract volumes from 51 subregions of the cerebellum, hippocampus, striatum, thalamus and globus pallidus(Chakravarty et al., 2006, Chakravarty et al., 2013, Park et al., 2014). The hippocampus was subdivided into 5 subregions (CA1, CA2/CA3, CA4/DG, SR/SL/SM and subiculum) (Pipitone et al., 2014, Winterburn et al., 2013). MAGeT processing and protocol details are described elsewhere (Chakravarty et al., 2013). A final quality control was carried out on the processed images, and one more participant with ADHD was removed due to failure (n = 141; ADHD = 106; control = 35).

### 2.4 Determination of cumulative exposure to ADHD medication

**Supplementary figure 1:**
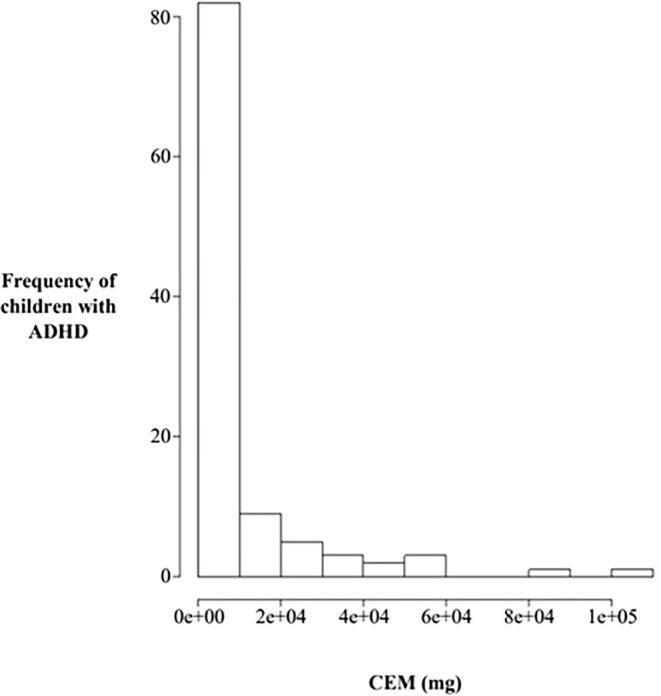


Lifetime pharmacological history of ADHD medication was collected retrospectively as reported by the parents, and subsequently corroborated against pharmacy prescription logs. All children with ADHD participating in the MRI study were exposed to medication for a minimum of one week prior to scanning (range 0.02 to 4.69 years, median = 0.25). Medication breaks (i.e., holidays, weekends and summer) were considered and subtracted from the total duration from the date of initial exposure to date of scanning. ADHD medications were prescribed by the treating psychiatrist at different doses for various durations depending on the clinical needs of the child. For each period of treatment at a given dose, the exposure to medication was calculated as the product of duration and dose. The cumulative exposure to ADHD medication (CEM) was then calculated by summing all the exposures (range 0.075 to 108.75 g, median = 1.5). Figure Supplemental Fig. 1 shows the untransformed distribution of CEM. In general, each child was prescribed a variety of medication brands, preventing the feasibility of a subgroup analysis for medication type. Dosage equivalencies between psychostimulant brands are comparable, apart from Adderall, which has double the potency of Ritalin. A total of 5 Adderall prescriptions were in our sample, and dosage was adjusted in a supplemental analysis. Moreover, a small subset of prescriptions was for the non-psychostimulant NE-specific agent, Strattera. A supplemental analysis controlling for Strattera exposure was conducted. Typically-developing children belonging to the control group had no exposure to ADHD medication.

### 2.5 Statistical analysis

#### 2.5.1 Children with ADHD

**Supplementary figure 2:**
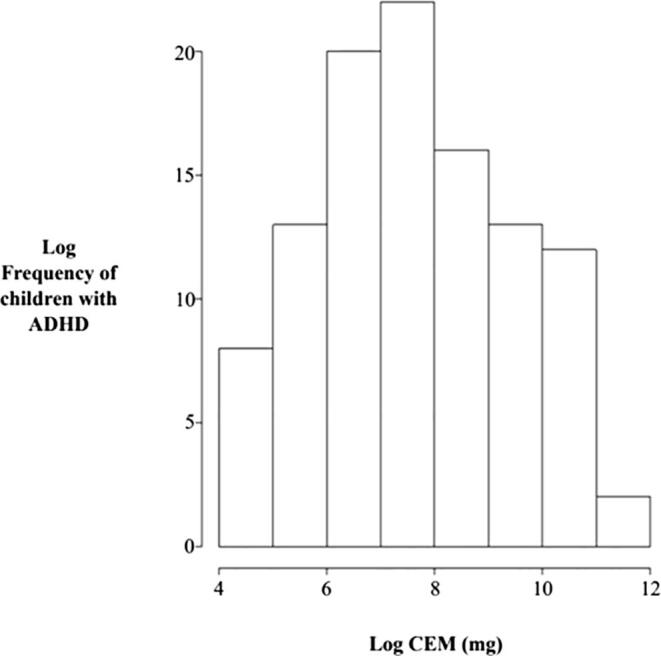


CEM data was log-transformed to generate a normal distribution (Supplemental Fig. 2) and RMINC was used to perform general linear modelling (GLM) (https://github.com/Mouse-Imaging-Centre/RMINC/). Age and sex were stated as covariates, and cortical thickness and surface area as main outcome measures. Similarly, a GLM was generated for analysis of 51 subcortical volumes and CEM, where age, sex and total brain volume were included as covariates, and subcortical volumes as main outcome measures. To address potential cofounds between CEM and ADHD severity, a supplemental analysis using the Conners’ Global Index scale (CGI), which is a measure of the child’s ADHD symptom severity at baseline was performed. Further, to explore the effect of medication on brain structure in relation to age, an interaction analysis between age and CEM was performed. A supplemental non-linear model (i.e., quadratic) was tested to explore the possibility of curvilinear effects of CEM on brain structure. Multiple-testing correction using FDR was performed.

To explore the relationship between brain structure and ADHD cognitive measures, significant findings were correlated with the Continuous Performance Test (CPT) dimensions. Four outcome measures (omissions, commissions, variability and reaction-time) were selected based on a *meta*-analysis associating them to ADHD(Huang-Pollock, Karalunas, Tam, & Moore, 2012). Correction for multiple comparisons was performed using Bonferroni for non-independent variables by considering the correlation between the 4 CPT measures (r = 0.43). The p-value cut-off was stated at 0.021.

#### 2.5.2 Group comparison between ADHD and control children

All analyses regarding CEM were conducted in a clinical sample of children being treated for ADHD, as typically-developing children are unmedicated. To explore whether the regions significantly associated with CEM were independent of ADHD diagnosis or age, two complimentary analyses using a control group (n = 35) were conducted. Subcortical volumes within 51 subregions were compared between cases and controls. Demographics were assessed between groups and measures that significantly differed were included as covariates in the model (i.e., sex and full-scale IQ). Specifically, age, sex, total brain volume, IQ and CEM were used as covariates, and each subcortical volume as the main outcome measure. Moreover, an interaction analysis between age and diagnosis was performed. Since a control group of typically-developing children exposed to ADHD medication is not ethically feasible, a 3-way interaction (age, ADHD diagnosis and CEM) was not possible to examine.

## 3 Results

### 3.1 Cumulative exposure to ADHD medication

Five children with ADHD were concurrently prescribed anti-psychotics and were excluded from the final analysis (ADHD n = 101). The number of independent prescriptions for ADHD medication per child was one (n = 7), two (n = 34), three (n = 21), four (n = 18) and five (n = 21). A total of 315 prescriptions were included: Ritalin® (35.2%), Biphentin® (32.4%), Concerta® (22.6%), Vyvanse® (5.7%), Strattera® (2.5%) and Adderall® (1.6%).

### 3.2 Cumulative exposure to ADHD medication and cortical structure

No global effects of CEM on cortical thickness or surface area were detected on either hemisphere (n = 101). CEM did not significantly predict cortical thickness and surface area measurements in the vertex-wise comparison at the set FDR range. Likewise, no effects of CEM on cortical structures were observed when controlling for ADHD severity.

### 3.3 Cumulative exposure to ADHD medication and subcortical volumes

**Fig. 1:**
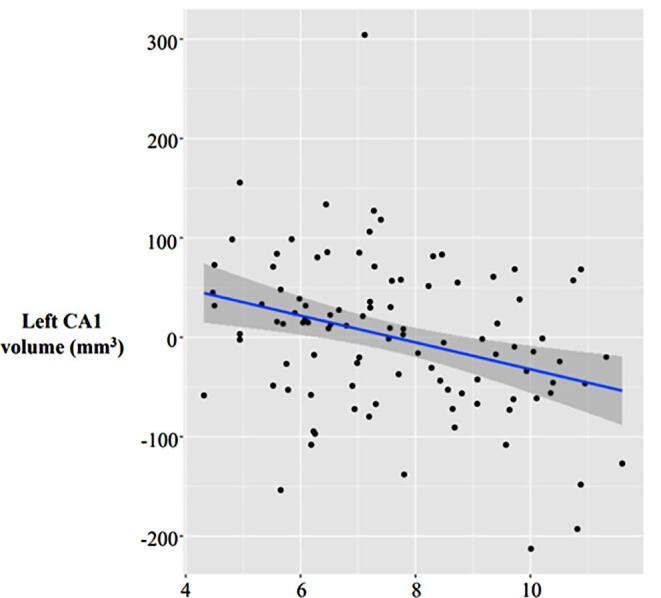
Graph representing association between cumulative exposure to ADHD medication and hippocampus CA1 volume.

Significant effects of CEM were found in 2 out of 5 subregions of the hippocampus, the left Cornu Ammonis 1 (CA1; df = 95; q = 0.003) and the left strata radiatum/lacunosum/moleculare (SR/SL/SM) (df = 95; q = 0.003).The right CA1 (df = 95; q = 0.06), right SR/SL/SM (df = 95; q = 0.08), right dentate gyrus (DG; df = 95; q = 0.08) and left CA2/3 (df = 95; q = 0.08) did not reach significance. Specifically, higher CEM was associated with decreased volumes within significant subregions (Fig. 1). Post-hoc analysis revealed an effect size of 38.5% at 99% power (predictors = 3; R-squared = 0.385; α = 0.05; n = 101). Moreover, the interaction analysis between CEM and age yielded no significant findings, and no significant effects were detected between hippocampus CA1 volumes and CPT dimensions.

Supplemental analyses controlling handedness and dosage equivalencies between medication types yielded the same findings (i.e., dextroamphetamine; Adderall®). Results remained significant after controlling for ADHD symptom severity (CA1 and SR/SL/SM; df = 94; q = 0.0147) as well as when controlling for exposure to the non-psychostimulant, atomoxetine (Strattera®) (CA1 and SR/SL/SM; df = 94; q = 0.006). Controlling for weight did not alter results. Moreover, the interaction analysis between CEM and age yielded no significant findings, and no significant effects were detected between hippocampus CA1 volumes and neuropsychiatric assessments (CPT performance). Finally, no non-linear effects of CEM were detected on the volumes of the 51 subregions included in our model.

### 3.4 Control children and subcortical volume

Age, income, ethnicity and handedness did not significantly differ between ADHD and control children. Significant group differences were found for sex and full-scale IQ, and thus were included as covariates in the analysis. As expected, significant differences in ADHD symptomatology and behavioural measures were detected between ADHD and control children (CBCL, Conners’, ADHD total items; Table 1). Group comparison between ADHD and control children revealed no significant volumetric differences within any of the 51 subregions. No significant effects were uncovered in the age-by-diagnosis interaction analysis.

## 4 Discussion

The goal of the present study was to investigate the effects of CEM, as defined by duration (days) and dose (mg/day), on cortical structures and subcortical volumes in medicated children with ADHD. To the best of our knowledge, this is the first neuroimaging study exploring the effects of CEM on 51 subcortical volumes in a sample of children all undergoing pharmacological treatment for ADHD. Significantly smaller volumes were found in subregions of the left hippocampus CA1 and SR/SL/SM and were associated with higher CEM. No significant medication effects were detected on cortical thickness or surface area.

Preceding neuroimaging studies have provided contradictory results concerning differences in hippocampus volume between ADHD and typically-developing children. A recent subcortical *meta*-analysis combining over 3000 scans from multiple sites reported smaller global hippocampus volumes in children with ADHD (Hoogman et al., 2017). This analysis was based on a mixed sample of treatment-naïve and chronically-treated children, and concluded that medication was not a contributing factor to the observed differences in subcortical volumes. However, the authors stated in their limitations that interpretation of results warrant some caution, as the study design was not optimal to test for medication effects. On the other hand, opposite findings have been reported, where children with ADHD were found to have larger hippocampus volumes compared to control children (Plessen et al., 2006). Sixty-nine percent of children were taking psychostimulants, and medication exposure was corrected in the analysis. Authors specified that the volume increase was driven by enlargement of the anterior region of the hippocampus, specifically the CA1, CA2/3 and DG subregions. Interestingly, the authors also observed contraction in the posterior portion of the hippocampus, indicating smaller volumes in the underlying tissues for the ADHD group within this region. These results suggest that the various hippocampus subregions may be differentially affected in ADHD pathophysiology and in parallel, differentially targeted by medication. However, few studies investigating the different hippocampus subregions in relation to ADHD exist in the literature. Al-Amin et al. reported reductions in several hippocampus subregions, including CA1, between ADHD and control children (n = 860; ADHD = 327; control = 533) (Al-Amin, Zinchenko, & Geyer, 2018), though, correction for medication exposure was not performed. Since a high proportion of children diagnosed with ADHD receive pharmacological treatment, it is possible that medication effects are confounding the findings on hippocampus subregions volumes between diagnostic groups (Al-Amin et al., 2018). The authors proposed that the volumetric reductions were caused by neuronal atrophy within the subregions (Al-Amin et al., 2018). Moreover, chronic methylphenidate (MPH) exposure at high doses has been shown to induce neuronal cell death in the rat hippocampus CA1 regions (Carvallo et al., 2018, Schmitz et al., 2017).

In the present study, the hippocampus was divided into five subregions (CA1, CA2/CA3, CA4/DG, SR/SL/SM and subiculum) and tested for association with CEM. Three supplemental analyses were conducted to help extrapolate medication effects: 1) case-control group comparison, and interaction analyses of 2) age-by-diagnosis and 3) age-by-CEM. Volumes within hippocampus subregions did not significantly differ between cases and controls in our sample, hinting that current findings were not unduly influenced by diagnostic effects. Previous case-control imaging studies that conveyed differences in hippocampus volumes used case groups either consisting solely of treatment-naïve ADHD children, or mixed exposure (i.e., never exposed, and exposed children, where status of medication exposure was not always considered) (Al-Amin et al., 2018, Hoogman et al., 2017). All the ADHD children in the present study have been exposed to medication for a minimum of 1 week, thereby controlling, at least partly, for heterogeneity of treatment exposure status in the analysis. Studies that specifically investigated medication effects on brain structure compared groups of treatment-naïve, treatment-exposed and typically-developing children, and failed to detect significant hippocampus volume differences between the exposed and control group (Nakao et al., 2011, Spencer et al., 2013). Furthermore, evidence exists for a delay in developmental trajectories in children with ADHD, with a larger magnitude of brain volume differences observed in children relative to adults (Hoogman et al., 2017, Shaw et al., 2018). The age-by-diagnosis analysis did not reveal any significant findings, suggesting that diagnostic effects on hippocampus subregions volumes, if existing in the first place, do not change as a function of age in our sample. Second, no significant effects were uncovered in the age-by-CEM interaction, suggesting that the effects of CEM on hippocampus subregions volumes remain stable with age. Taken together, current findings propose that the association between CEM and hippocampus subregions volumes is independent of age and ADHD diagnosis.

Pharmacological studies conducted in rodents have found that psychostimulants increased synaptic levels of DA and NE in several brain regions, notably the prefrontal cortex, a region robustly associated with ADHD, and the hippocampus (Berridge and Devilbiss, 2011, Carvallo et al., 2018, Kuczenski and Segal, 2001). Changes in the hippocampus induced by psychostimulants have been implicated in the therapeutic response and as a potential side-effect (Britton & Bethancourt, 2009). MPH has been shown to increase DA/NE concentrations in a dose-dependent fashion within the hippocampus of adolescent rats, and thus MPH exposure has been proposed to impact hippocampus development (Kuczenski and Segal, 2002, Schmitz et al., 2017). Santos et al. reported that chronic MPH treatment administered to control rats caused synaptic remodelling within the hippocampus, which led to memory and cognitive deficits (Coelho-Santos et al., 2019). A study comparing effects of low versus high doses of chronic MPH treatment on hippocampus cell proliferation and survival found that administration at both doses increased neurogenesis. However, the maintenance and integration of newly-formed neurons were only observed in the low-dose group. Authors concluded that chronic exposure to MPH at high doses initially increased neurogenesis but that hippocampus atrophy ensued as newly-formed neurons failed to survive long-term (Oakes et al., 2018). A review on the neurotoxic effects of psychostimulants reported similar findings, where young rats repeatedly administered MPH displayed significant decreases in the number of neurons and astrocytes in the hippocampus. However, neuronal proliferation was not affected by MPH (Goncalves, Baptista, & Silva, 2014). Furthermore, chronic MPH treatment at high doses has been linked with oxidative stress, neuroinflammation and neurodegeneration in the hippocampus of rats (Motaghinejad, Motevalian, & Shabab, 2016). Indeed, early and chronic administration of MPH was shown to ultimately cause deformations in the shape of the rat hippocampus (Coelho-Santos et al., 2019, van der Marel et al., 2015).

Nevertheless, the studies highlighted above employed non-ADHD animal models with typical catecholamine functioning. Since the current model for ADHD pathophysiology consists of DA/NE dysregulation, medication is likely to differently impact the brain of ADHD individuals (Biezonski et al., 2016). Convergent evidence exists of a dose-dependent U-shape therapeutic response from MPH, where lower doses improve cognitive performance and higher doses induce neurotoxic effects and cognitive impairment by bringing DA/NE above optimal concentrations (Cheng et al., 2014, Coelho-Santos et al., 2019, Devilbiss and Berridge, 2008). In the current study, smaller hippocampus subregion volumes were associated with higher CEM. Future studies are required to determine whether the nature of these alterations are therapeutic benefits or side-effects induced by continual exposure to ADHD medication use in humans.

Although it is uncertain if deficits in neurogenesis and long-term memory are involved in ADHD pathophysiology, the hippocampus is also involved in motivation and emotional regulation, which are functions impaired in ADHD individuals. Although the underlying cause for the volumetric reductions in the present study cannot be confirmed, it is reasonable to hypothesize, given the considerable evidence from animal studies, that alterations in neurogenesis and synaptic modelling following chronic exposure to ADHD medication are driving the structural changes observed. In addition, no significant associations with omissions, commissions, variability, and reaction-time CPT scores were detected, suggesting that selective and sustained attention was not related to the CA1 volume reduction in our sample. However, further research is required to determine whether these volumetric decreases are directly associated with long-term memory function, motivation, emotional regulation, or other behaviours mediated by the hippocampus in humans.

The major strength of this study is the detailed variable created for CEM. The structured interview with the parents in conjunction with access to the child’s prescription history provides the information required to generate a precise and quantitative value of medication exposure, which considers dose, duration, multiple prescriptions, and medication breaks. External validation of parental reports was also made possible through prescription pharmacy logs. The third strength is the specificity of the subcortical regions under study, which were subdivided into 51 regions to explore more localized effects, notably in the hippocampus. Moreover, restricting the sample to medicated children shifted the focus from comparing groups of treatment-naïve to treatment-exposed children with ADHD, to investigating medication effects within continuously-treated children with ADHD. This enabled the possibility of examining the association between various cumulative exposure points and subregional volumes, instead of assessing the effects initial exposure to medication between groups. Another strength is that all ADHD children were undergoing treatment for ADHD, which helped diminish motion during scanning and yielded viable scans that passed quality control (i.e., apart from 3 children with ADHD). Furthermore, all scans were carried out on the same scanner located on site at the DMHUI, removing multi-site error.

The present study should be viewed in light of its limitations. First, the cross-sectional design prevented the assessment of medication effects over time on brain development. Longitudinal studies investigating brain development as a function of CEM are warranted. Differences in brain structure are prominent in children, and while the effects of age were accounted for in the model, our sample was limited to a pediatric population limiting the generalizability of findings to other age groups. Similarly, sex was included in the model, though it is plausible that medication effects on brain structure are divergent in boys and girls. Previous studies in humans (Griffin et al., 1989, Robbins et al., 1999) and in animals (Becker, 2016) have shown that females have a higher sensitivity to stimulants such as cocaine relative to males, highlighting the importance of future studies investigating sex difference in the relationship between CEM and brain structure. We did not control for comorbidity in this study, however children on medications for treatment other than ADHD were excluded from the analysis (n = 5). Although most prescriptions in our cohort were psychostimulants (97.5%), we cannot entirely discount the potential differences between medication brands. Nevertheless, dosage equivalencies among psychostimulant brands are relatively similar, apart from dextroamphetamine (Adderall®). Dosage was adjusted in a supplemental analysis and yielded the same findings. Furthermore, a supplemental analysis controlling for atomoxetine exposure (Strattera®), a non-psychostimulant, was conducted and CEM effects on left CA1 and SR/SL/SM volumes remained significant (df = 94; q = 0.006). Fourth, while the sample size (n = 101) was sufficiently powered to uncover medication effects on hippocampus subregions, repeating the analysis in a larger independent cohort is required to confirm these findings, as well as increase power to detect potential smaller medication effects in other brain regions. This can be extended to the modest sample size of control children in this study, which produced no significant results against the ADHD treatment group.

## 5 Conclusions

The therapeutic response of ADHD medication and associated side-effects on behaviour are well documented in the literature. However, the effects of prolonged ADHD medication use and dosage on human brain structure remain elusive. This knowledge gap is relevant to researchers and healthcare providers, and raises important concern for individuals taking ADHD medication and parents of children with ADHD. Here, it was found that higher CEM was associated with smaller hippocampus volumes in the CA1 and SR/SL/SM subregions in medicated children with ADHD, and that these effects were independent of ADHD severity, sex, and age. Despite extensive research, neuroimaging studies in ADHD have garnered contradictory and irreproducible findings. This may be partly attributable to unaccounted medication effects on brain structure and head movements during scanning. Therefore, our results suggest that the effects of CEM should be considered in future ADHD neuroimaging research. Furthermore, awareness of the structural consequences induced by medication on the hippocampus sheds light on the pathophysiology of ADHD and may influence the decision-making process of ADHD treatment in children. Although smaller hippocampus subregional volumes were not associated with the cognitive dimensions tested in our sample, previous research has shown that smaller hippocampus volumes are associated with increased vulnerability to brain disorders later in life, memory deficits, sensitivity to trauma and anti-depressant resistance. Therefore, understanding the effects of CEM on the hippocampus may be an important factor in determining the optimal duration and dosage of treatment to avoid negative life outcomes, which may in turn instigate revaluation of current ADHD prescription practices. In summary, we report on a thorough approach to quantify cumulative medication effects and show smaller subcortical volumes are associated with higher cumulative exposure. This effect remained even when accounting for age and severity of symptoms. Although these findings are interesting, they should be interpreted with caution given the study’s limited cross-sectional design.

## CRediT authorship contribution statement

**Nellie H. Fotopoulos:** Conceptualization, Investigation, Methodology, Formal analysis, Visualization, Writing - review & editing. **Gabriel A. Devenyi:** Methodology, Software, Data curation, Resources. **Stephanie Guay:** Data curation. **Sarojini M. Sengupta:** Supervision. **M. Mallar Chakravarty:** Resources, Software. **Natalie Grizenko:** Resources, Funding acquisition. **Sherif Karama:** Supervision, Validation, Methodology, Writing - review & editing. **Ridha Joober:** Supervision, Resources, Funding acquisition, Writing - review & editing.

## Declaration of Competing Interest

The authors declare that they have no known competing financial interests or personal relationships that could have appeared to influence the work reported in this paper.

## References

[b0005] Ad-Dab'bagh Y., Einarson D., Lyttelton O., Muehlboeck J.-S., Mok K., Ivanov O. (2006). The civet image-processing environment: a fully automated comprehensive pipeline for anatomical Neuroimaging research. Proceedings of the 12th Annual Meeting of the Organization for Human Brain Mapping.

[b0010] Al-Amin M., Zinchenko A., Geyer T. (2018). Hippocampal subfield volume changes in subtypes of attention deficit hyperactivity disorder. Brain Res.

[b0015] Albajara Saenz A., Villemonteix T., Massat I. (2018). Structural and functional neuroimaging in attention-deficit/hyperactivity disorder. Dev Med Child Neurol.

[b0020] Atkinson M., Hollis C. (2010). NICE guideline: attention deficit hyperactivity disorder. Arch Dis Child Educ Pract Ed.

[b0025] Becker J.B. (2016). Sex differences in addiction. Dialogues Clin Neurosci.

[b0030] Berridge C.W., Devilbiss D.M. (2011). Psychostimulants as cognitive enhancers: the prefrontal cortex, catecholamines, and attention-deficit/hyperactivity disorder. Biol Psychiatry.

[b0035] Biezonski, D., Shah, R., Krivko, A., Cha, J., Guilfoyle, D. N., Hrabe, J., . . . Posner, J. (2016). Longitudinal magnetic resonance imaging reveals striatal hypertrophy in a rat model of long-term stimulant treatment. Transl Psychiatry, 6(9), e884. 10.1038/tp.2016.158. 10.1038/tp.2016.158 PMC5048200 27598968

[b0040] Bledsoe J., Semrud-Clikeman M., Pliszka S.R. (2009). A magnetic resonance imaging study of the cerebellar vermis in chronically treated and treatment-naive children with attention-deficit/hyperactivity disorder combined type. Biol Psychiatry.

[b0045] Briars L., Todd T. (2016). A Review of Pharmacological Management of Attention-Deficit/Hyperactivity Disorder. J Pediatr Pharmacol Ther.

[b0050] Britton G.B., Bethancourt J.A. (2009). Characterization of anxiety-related responses in male rats following prolonged exposure to therapeutic doses of oral methylphenidate. Pharmacol Biochem Behav.

[b0055] Carvallo C., Contreras D., Ugarte G., Delgado R., Pancetti F., Rozas C., Piña R., Constandil L., Zeise M.L., Morales B. (2018). Single and Repeated Administration of Methylphenidate Modulates Synaptic Plasticity in Opposite Directions via Insertion of AMPA Receptors in Rat Hippocampal Neurons. Front Pharmacol.

[b0060] Castellanos F.X., Lee P.P., Sharp W., Jeffries N.O., Greenstein D.K., Clasen L.S., Rapoport J.L. (2002). Developmental trajectories of brain volume abnormalities in children and adolescents with attention-deficit/hyperactivity disorder. JAMA.

[b0065] Chakravarty M.M., Bertrand G., Hodge C.P., Sadikot A.F., Collins D.L. (2006). The creation of a brain atlas for image guided neurosurgery using serial histological data. Neuroimage.

[b0070] Chakravarty M.M., Steadman P., van Eede M.C., Calcott R.D., Gu V., Shaw P., Raznahan A., Collins D.L., Lerch J.P. (2013). Performing label-fusion-based segmentation using multiple automatically generated templates. Hum Brain Mapp.

[b0075] Cheng J., Xiong Z., Duffney L.J., Wei J., Liu A., Liu S., Chen G.-J., Yan Z. (2014). Methylphenidate exerts dose-dependent effects on glutamate receptors and behaviors. Biol Psychiatry.

[b0080] Coelho-Santos V., Cardoso F.L., Magalhães A., Ferreira-Teixeira M., Leitão R.A., Gomes C., Rito M., Barbosa M., Fontes-Ribeiro C.A., Silva A.P. (2019). Effect of chronic methylphenidate treatment on hippocampal neurovascular unit and memory performance in late adolescent rats. Eur Neuropsychopharmacol.

[b0085] Collins D.L., Holmes C.J., Peters T.M., Evans A.C. (1995). Automatic 3-D model-based neuroanatomical segmentation. Hum Brain Mapp.

[b0090] Conners C.K. (1985). The computerized continuous performance test. Psychopharmacol Bull.

[b0095] Conners C.K., Sitarenios G., Parker J.D., Epstein J.N. (1998). Revision and restandardization of the Conners Teacher Rating Scale (CTRS-R): factor structure, reliability, and criterion validity. J Abnorm Child Psychol.

[b0100] Danielson, M. L., Bitsko, R. H., Ghandour, R. M., Holbrook, J. R., Kogan, M. D., & Blumberg, S. J. (2018). Prevalence of Parent-Reported ADHD Diagnosis and Associated Treatment Among U.S. Children and Adolescents, 2016. J Clin Child Adolesc Psychol, 47(2), 199-212. 10.1080/15374416.2017.1417860. 10.1080/15374416.2017.1417860 PMC5834391 29363986

[b0105] Devilbiss D.M., Berridge C.W. (2008). Cognition-enhancing doses of methylphenidate preferentially increase prefrontal cortex neuronal responsiveness. Biol Psychiatry.

[b0110] Diagnostic and Statistical Manual of Mental Disorders. (1994). (4th ed.). Washington, DC.

[b0115] Faraone S.V. (2018). The pharmacology of amphetamine and methylphenidate: Relevance to the neurobiology of attention-deficit/hyperactivity disorder and other psychiatric comorbidities. Neurosci Biobehav Rev.

[b0120] Faraone S.V., Asherson P., Banaschewski T., Biederman J., Buitelaar J.K., Ramos-Quiroga J.A., Rohde L.A., Sonuga-Barke E.J.S., Tannock R., Franke B. (2015). Attention-deficit/hyperactivity disorder. Nat Rev Dis Primers.

[b0125] Franke, B., Michelini, G., Asherson, P., Banaschewski, T., Bilbow, A., Buitelaar, J. K., . . . Reif, A. (2018). Live fast, die young? A review on the developmental trajectories of ADHD across the lifespan. Eur Neuropsychopharmacol. 10.1016/j.euroneuro.2018.08.001. 10.1016/j.euroneuro.2018.08.001 PMC6379245 30195575

[b0130] Frodl T., Skokauskas N. (2012). Meta-analysis of structural MRI studies in children and adults with attention deficit hyperactivity disorder indicates treatment effects. Acta Psychiatr Scand.

[b0135] Goncalves J., Baptista S., Silva A.P. (2014). Psychostimulants and brain dysfunction: a review of the relevant neurotoxic effects. Neuropharmacology.

[b0140] Greven C.U., Bralten J., Mennes M., O’Dwyer L., van Hulzen K.J.E., Rommelse N., Schweren L.J.S., Hoekstra P.J., Hartman C.A., Heslenfeld D., Oosterlaan J., Faraone S.V., Franke B., Zwiers M.P., Arias-Vasquez A., Buitelaar J.K. (2015). Developmentally stable whole-brain volume reductions and developmentally sensitive caudate and putamen volume alterations in those with attention-deficit/hyperactivity disorder and their unaffected siblings. JAMA Psychiatry.

[b0145] Griffin M.L., Weiss R.D., Mirin S.M., Lange U. (1989). A comparison of male and female cocaine abusers. Arch Gen Psychiatry.

[b0150] Hoogman M., Bralten J., Hibar D.P., Mennes M., Zwiers M.P., Schweren L.S.J., van Hulzen K.J.E., Medland S.E., Shumskaya E., Jahanshad N., Zeeuw P.d., Szekely E., Sudre G., Wolfers T., Onnink A.M.H., Dammers J.T., Mostert J.C., Vives-Gilabert Y., Kohls G., Oberwelland E., Seitz J., Schulte-Rüther M., Ambrosino S., Doyle A.E., Høvik M.F., Dramsdahl M., Tamm L., van Erp T.G.M., Dale A., Schork A., Conzelmann A., Zierhut K., Baur R., McCarthy H., Yoncheva Y.N., Cubillo A., Chantiluke K., Mehta M.A., Paloyelis Y., Hohmann S., Baumeister S., Bramati I., Mattos P., Tovar-Moll F., Douglas P., Banaschewski T., Brandeis D., Kuntsi J., Asherson P., Rubia K., Kelly C., Martino A.D., Milham M.P., Castellanos F.X., Frodl T., Zentis M., Lesch K.-P., Reif A., Pauli P., Jernigan T.L., Haavik J., Plessen K.J., Lundervold A.J., Hugdahl K., Seidman L.J., Biederman J., Rommelse N., Heslenfeld D.J., Hartman C.A., Hoekstra P.J., Oosterlaan J., Polier G.V., Konrad K., Vilarroya O., Ramos-Quiroga J.A., Soliva J.C., Durston S., Buitelaar J.K., Faraone S.V., Shaw P., Thompson P.M., Franke B. (2017). Subcortical brain volume differences in participants with attention deficit hyperactivity disorder in children and adults: a cross-sectional mega-analysis. Lancet Psychiatry.

[b0155] Hoogman M., van Rooij D., Klein M., Boedhoe P., Ilioska I., Li T., Franke B. (2020). Consortium neuroscience of attention deficit/hyperactivity disorder and autism spectrum disorder: The ENIGMA adventure. Hum Brain Mapp.

[b0160] Huang-Pollock C.L., Karalunas S.L., Tam H., Moore A.N. (2012). Evaluating vigilance deficits in ADHD: a meta-analysis of CPT performance. J Abnorm Psychol.

[b0165] Ivanov I., Bansal R., Hao X., Zhu H., Kellendonk C., Miller L., Sanchez-Pena J., Miller A.M., Chakravarty M.M., Klahr K., Durkin K., Greenhill L.L., Peterson B.S. (2010). Morphological abnormalities of the thalamus in youths with attention deficit hyperactivity disorder. Am J Psychiatry.

[b0170] Kasius M.C., Ferdinand R.F., Berg H., Verhulst F.C. (1997). Associations between different diagnostic approaches for child and adolescent psychopathology. J Child Psychol Psychiatry.

[b0175] Klein R.G., Mannuzza S., Olazagasti M.A., Roizen E., Hutchison J.A., Lashua E.C., Castellanos F.X. (2012). Clinical and functional outcome of childhood attention-deficit/hyperactivity disorder 33 years later. Arch Gen Psychiatry.

[b0180] Kuczenski R., Segal D.S. (2001). Locomotor effects of acute and repeated threshold doses of amphetamine and methylphenidate: relative roles of dopamine and norepinephrine. J Pharmacol Exp Ther.

[b0185] Kuczenski R., Segal D.S. (2002). Exposure of adolescent rats to oral methylphenidate: preferential effects on extracellular norepinephrine and absence of sensitization and cross-sensitization to methamphetamine. J Neurosci.

[b0190] Loureiro-Vieira S., Costa V.M., de Lourdes Bastos M., Carvalho F., Capela J.P. (2017). Methylphenidate effects in the young brain: friend or foe?. Int J Dev Neurosci.

[b0195] Lukito S., Norman L., Carlisi C., Radua J., Hart H., Simonoff E., Rubia K. (2020). Comparative meta-analyses of brain structural and functional abnormalities during cognitive control in attention-deficit/hyperactivity disorder and autism spectrum disorder. Psychol Med.

[b0200] Motaghinejad M., Motevalian M., Shabab B. (2016). Effects of chronic treatment with methylphenidate on oxidative stress and inflammation in hippocampus of adult rats. Neurosci Lett.

[b0205] Nakao T., Radua J., Rubia K., Mataix-Cols D. (2011). Gray matter volume abnormalities in ADHD: voxel-based meta-analysis exploring the effects of age and stimulant medication. Am J Psychiatry.

[b0210] Norman L.J., Carlisi C., Lukito S., Hart H., Mataix-Cols D., Radua J., Rubia K. (2016). Structural and Functional Brain Abnormalities in Attention-Deficit/Hyperactivity Disorder and Obsessive-Compulsive Disorder: A Comparative Meta-analysis. JAMA Psychiatry.

[b0215] Oakes H.V., DeVee C.E., Farmer B., Allen S.A., Hall A.N., Ensley T., Pond B.B. (2018). Neurogenesis within the hippocampus after chronic methylphenidate exposure. J Neural Transm (Vienna).

[b0220] Onnink A.M., Zwiers M.P., Hoogman M., Mostert J.C., Kan C.C., Buitelaar J., Franke B. (2014). Brain alterations in adult ADHD: effects of gender, treatment and comorbid depression. Eur Neuropsychopharmacol.

[b0225] Pardoe H.R., Kucharsky Hiess R., Kuzniecky R. (2016). Motion and morphometry in clinical and nonclinical populations. Neuroimage.

[b0230] Park M.T.M., Pipitone J., Baer L.H., Winterburn J.L., Shah Y., Chavez S., Schira M.M., Lobaugh N.J., Lerch J.P., Voineskos A.N., Chakravarty M.M. (2014). Derivation of high-resolution MRI atlases of the human cerebellum at 3T and segmentation using multiple automatically generated templates. Neuroimage.

[b0235] Pipitone J., Park M.T.M., Winterburn J., Lett T.A., Lerch J.P., Pruessner J.C., Lepage M., Voineskos A.N., Chakravarty M.M. (2014). Multi-atlas segmentation of the whole hippocampus and subfields using multiple automatically generated templates. Neuroimage.

[b0240] Plessen K.J., Bansal R., Zhu H., Whiteman R., Amat J., Quackenbush G.A., Martin L., Durkin K., Blair C., Royal J., Hugdahl K., Peterson B.S. (2006). Hippocampus and amygdala morphology in attention-deficit/hyperactivity disorder. Arch Gen Psychiatry.

[b0245] Pretus C., Ramos-Quiroga J.A., Richarte V., Corrales M., Picado M., Carmona S., Vilarroya O. (2017). Time and psychostimulants: Opposing long-term structural effects in the adult ADHD brain. A longitudinal MR study. *Eur Neuropsychopharmacol*.

[b0250] Prevention., C. f. D. C. a. (September 21st 2018). Data and Statistics About ADHD. Retrieved from https://www.cdc.gov/ncbddd/adhd/data.html.

[b0255] Reuter M., Tisdall M.D., Qureshi A., Buckner R.L., van der Kouwe A.J.W., Fischl B. (2015). Head motion during MRI acquisition reduces gray matter volume and thickness estimates. Neuroimage.

[b0260] Robbins S.J., Ehrman R.N., Childress A.R., O'Brien C.P. (1999). Comparing levels of cocaine cue reactivity in male and female outpatients. Drug Alcohol Depend.

[b0265] Rubia K., Alegria A.A., Cubillo A.I., Smith A.B., Brammer M.J., Radua J. (2014). Effects of stimulants on brain function in attention-deficit/hyperactivity disorder: a systematic review and meta-analysis. Biol Psychiatry.

[b0270] Schmitz F., Pierozan P., Rodrigues A.F., Biasibetti H., Grunevald M., Pettenuzzo L.F., Scaini G., Streck E.L., Netto C.A., Wyse A.T.S. (2017). Methylphenidate Causes Behavioral Impairments and Neuron and Astrocyte Loss in the Hippocampus of Juvenile Rats. Mol Neurobiol.

[b0275] Schweren L.J., de Zeeuw P., Durston S. (2013). MR imaging of the effects of methylphenidate on brain structure and function in attention-deficit/hyperactivity disorder. Eur Neuropsychopharmacol.

[b0280] Schweren L.J.S., Hartman C.A., Zwiers M.P., Heslenfeld D.J., van der Meer D., Franke B., Oosterlaan J., Buitelaar J.K., Hoekstra P.J. (2015). Combined stimulant and antipsychotic treatment in adolescents with attention-deficit/hyperactivity disorder: a cross-sectional observational structural MRI study. Eur Child Adolesc Psychiatry.

[b0285] Semrud-Clikeman M., Pliszka S.R., Bledsoe J., Lancaster J. (2014). Volumetric MRI differences in treatment naive and chronically treated adolescents with ADHD-combined type. J Atten Disord.

[b0290] Sengupta S.M., Fotopoulos N., Devenyi G.A., Fortier M.-È., Ter-Stepanian M., Sagliker S., Karama S., Mallar Chakravarty M., Labbe A., Grizenko N., Joober R. (2018). Dissecting genetic cross-talk between ADHD and other neurodevelopmental disorders: evidence from behavioural, pharmacological and brain imaging investigations. Psychiatry research.

[b0295] Shaw P., De Rossi P., Watson B., Wharton A., Greenstein D., Raznahan A., Sharp W., Lerch J.P., Chakravarty M.M. (2014). Mapping the development of the basal ganglia in children with attention-deficit/hyperactivity disorder. J Am Acad Child Adolesc Psychiatry.

[b0300] Shaw P., Eckstrand K., Sharp W., Blumenthal J., Lerch J.P., Greenstein D., Clasen L., Evans A., Giedd J., Rapoport J.L. (2007). Attention-deficit/hyperactivity disorder is characterized by a delay in cortical maturation. Proc Natl Acad Sci U S A.

[b0305] Shaw P., Ishii-Takahashi A., Park M.T., Devenyi G.A., Zibman C., Kasparek S., Sudre G., Mangalmurti A., Hoogman M., Tiemeier H., von Polier G., Shook D., Muetzel R., Chakravarty M.M., Konrad K., Durston S., White T. (2018). A multicohort, longitudinal study of cerebellar development in attention deficit hyperactivity disorder. J Child Psychol Psychiatry.

[b0310] Shaw P., Sharp W.S., Morrison M., Eckstrand K., Greenstein D.K., Clasen L.S., Evans A.C., Rapoport J.L. (2009). Psychostimulant treatment and the developing cortex in attention deficit hyperactivity disorder. Am J Psychiatry.

[b0315] Sled J.G., Zijdenbos A.P., Evans A.C. (1998). A nonparametric method for automatic correction of intensity nonuniformity in MRI data. IEEE Trans Med Imaging.

[b0320] Spencer T.J., Brown A., Seidman L.J., Valera E.M., Makris N., Lomedico A., Faraone S.V., Biederman J. (2013). Effect of psychostimulants on brain structure and function in ADHD: a qualitative literature review of magnetic resonance imaging-based neuroimaging studies. J Clin Psychiatry.

[b0325] van der Marel K., Bouet V., Meerhoff G.F., Freret T., Boulouard M., Dauphin F., Klomp A., Lucassen P.J., Homberg J.R., Dijkhuizen R.M., Reneman L. (2015). Effects of long-term methylphenidate treatment in adolescent and adult rats on hippocampal shape, functional connectivity and adult neurogenesis. Neuroscience.

[b0330] Weinberger D.R., Radulescu E. (2016). Finding the Elusive Psychiatric “Lesion” With 21st-Century Neuroanatomy: A Note of Caution. Am J Psychiatry.

[b0335] Winterburn J.L., Pruessner J.C., Chavez S., Schira M.M., Lobaugh N.J., Voineskos A.N., Chakravarty M.M. (2013). A novel in vivo atlas of human hippocampal subfields using high-resolution 3 T magnetic resonance imaging. Neuroimage.

